# The effect of the family presence on anxiety and agitation of patients under mechanical ventilation after open heart surgery: a randomized clinical trial

**DOI:** 10.1186/s13741-021-00207-2

**Published:** 2021-11-01

**Authors:** Jamileh Mokhtari Nouri, Leila Safaeipour, Zohreh Vafadar, Seyed Tayeb Moradian

**Affiliations:** 1 Quran and Hadith Research Center, Nursing Faculty, Baqiyatallah University of Medical Sciences, Tehran, Iran; 2 Baqiyatallah University of Medical Sciences, Tehran, Iran; 3 Health Management Research Center, Baqiyatallah University of Medical Sciences, Tehran, Iran; 4 Atherosclerosis Research Center, Baqiyatallah University of Medical Sciences, Tehran, Iran

**Keywords:** Anxiety, Coronary artery bypass surgery, Critical care, Family-centered nursing, Psychomotor agitation, Weaning

## Abstract

**Background:**

Family-centered care has been considered as a philosophy of care. Family presence in intensive care units (ICUs), especially in the acute phase of the disease is controversial. This study has been carried out in order to determine the effect of the family presence on anxiety and agitation in patients undergoing coronary artery bypass grafting (CABG).

**Materials and methods:**

In a clinical trial, 70 patients were randomly allocated into groups of experimental and control. In the experimental group, during the weaning process from the mechanical ventilation, a family member was present at the bedside. The degree of anxiety and Richmond’s Agitation and Sedation Scale (RASS) were compared in seven consecutive time stages, including the time of entry into the ICU, the first respiratory drive, the family entrance, 20 min and 1 h after the presence of the family member, the time of extubation, and 1 h after extubation.

**Results:**

There was a significant difference between the two groups in the mean scores of the anxiety scale in the first (*P* =0.008), second (*P*=0.002), and third stages (*P* =0.005). This difference was not significant in the fourth to seventh stages (*P*>0.05). As the baseline anxiety levels were different, a covariate adjustment was used for comparisons between treatments, adjusting the main analyses for baseline anxiety levels. Analysis showed that groups were not different. Also, there was no significant difference in the mean scores of RASS between the two experimental and control groups at any of the seven stages (*P*> 0.05).

**Conclusion:**

According to the findings of the present study, the presence of a family member does not reduce the level of anxiety and agitation of patients undergoing cardiac surgery. However, it can be concluded that this intervention is feasible in acute and complex situations after open heart surgeries.

**Trial registration:**

This study has been registered in the Iranian Registry of Clinical Trials with the code IRCT201609014299N4.

## Background

In recent years, the number and quality of cardiac surgeries have increased (Thourani et al. 2017). After surgery, patients are transferred to the ICU, while they are monitored and mechanically ventilated. Mechanically ventilated patients experience numerous physiological and psychological complications (Chlan 2002; Wojnicki-Johansson 2001). Patients respond to stressful stimuli in three physiological, behavioral, and psychological dimensions (Tate et al. 2012). Anxiety is common after cardiac surgery (Aghaie et al. 2014; Page et al. 2017) and has been reported from even the moment being a candidate for surgery until discharge (Kalogianni et al. 2016; Korbmacher et al. 2013). Agitation leads to increased oxygen demand and consumption, hospital stay, and unplanned catheter removal (Cohen et al. 2002; Jaber et al. 2005).

Family visits are one of the most suggested interventions for ICU patients (Pun et al. 2019). The results of previous studies about the effect of the family presence on procedural anxiety are controversial. Some believe that the presence of family members reduces patients’ anxiety and agitation (Shapira and Tamir 1996; Kamali et al. 2020; Çelik et al. 2013). Others have not reported any benefit from the family presence (İşlekdemir and Kaya 2016; Sağlık and Çağlar 2019). Also, studies regarding the family presence after cardiac surgery are limited. Family visits in ICUs are conducted with three different policies: no-visit, limited visit, and open visit at all hours (da Silva Ramos et al. 2014). There is no equal policy between different countries and even the medical centers of a country. In countries such as Sweden, free visits are reported in 70% of centers and less than 1% in Italy (Liu et al. 2013; Cappellini et al. 2014). In Iran, there is no clear guideline for hospital visits, especially in ICUs. The family presence in the ICUs is often challenged. Many intensive care nurses are worried about the possible detrimental effects of visitation on patients (Adams et al. 2011; Sims and Miracle 2006). Also, in most centers with a flexible visitation policy, the presence of the family is prevented in the acute phase of the disease (Berti et al. 2007). Some studies have reported an increase in workload and delay in performing some medical interventions during family visits (Biancofiore et al. 2010; Slota et al. 2003). Evidence for increasing the infection rate in this area is lacking, and the family presence limitation reflects nurses’ personal preferences and concerns (Smith et al. 2009). It is said that nurses often prevent the presence of families, despite knowing the benefits (Biancofiore et al. 2010). Therefore, due to the high prevalence of anxiety and agitation in the acute phase after cardiac surgery, the present study has aimed to determine the effect of the family presence on anxiety and agitation in patients undergoing CABG surgery.

## Material and methods

### Study design and participants

This single-blinded randomly allocated parallel-group study (IRCT201404096778N5) was conducted from February to December 2016 in a university hospital. Totally, 72 patients who were undergoing cardiac surgery were randomly allocated into groups of intervention and control, each consisting of 36 participants. The randomization sequence was created using Excel 2007 (Microsoft, Redmond, WA, USA) with a 1:1 allocation, using random block sizes of 2 and 4 by an investigator with no clinical involvement in the trial. The details of the series were unknown to all investigators and coordinators. After the nurse had obtained the patient’s consent, she/he telephoned a contact independent of the recruitment process for allocation consignment.

### Inclusion and exclusion criteria

Patients with a negative history of psychological disorders, non-emergency surgeries, ejection fraction more than 30%, and non-redo surgery were included. The exclusion criteria were drainage of more than 400 mL during the first 4 h after surgery, severe hemodynamic instability, neurologic complications, and requiring mechanical ventilation more than 24 h after the surgery.

### Sample size

By using the Altman nomogram for a two-sided hypothesis with a power of 80% and *α*=0.05, the sample size was calculated at 32 people for each group. Considering a 10% attrition rate, the required sample size for this study was 72 patients. The sample size was calculated based on the anxiety variable and the standardized difference for this variable which was 1.20 (Kim and Lee 2015).

### Ethic

The ethics committee of a medical sciences university approved the study protocol (BMSU7191). One day before the operation, the study objectives were explained to the participants and informed consent was obtained.

### Procedure during surgery and in ICU

Surgical and anesthesia protocols were similar in both groups. The anesthesia was induced and maintained using midazolam, fentanyl, and propofol, and paralysis was achieved by atracurium. Patients were intubated after induction and ventilated during the surgery. The surgery was conducted using a standard procedure through a median sternotomy.

**Fig. 1:**
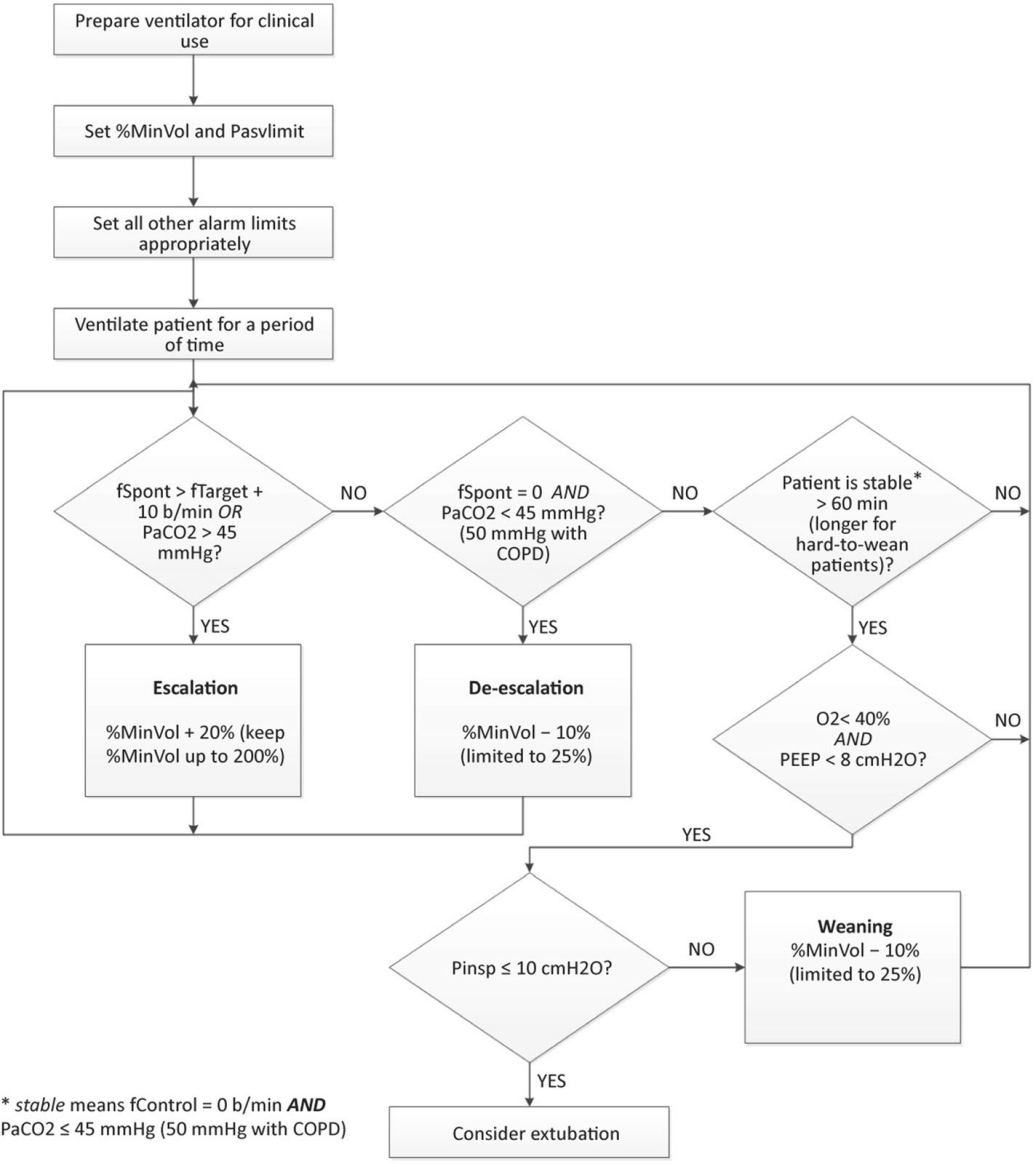
Management of mechanical ventilation using ASV mode

After the surgery, patients were transferred to the ICU and managed by experienced nurses. All patients were ventilated using a Hamilton GS ventilator. A Galileo with the software version GBC 01.202 (Hamilton Medical AG, Rhäzüns, Switzerland, software version 2.1X) was used for managing the patients during the mechanical ventilation. The Adaptive Support Ventilation (ASV) technology is fully described elsewhere. In both groups, the initial ventilator settings were minute volume 100%, oxygen inspiratory fraction (FiO_2_) of 100%, positive end-expiratory pressure of 5 cm H_2_O, and flow trigger sensitivity of 4 l/min. After 10 min, an arterial blood gas (ABG) analysis was performed, and based on the results, the settings were managed. The management of the ASV mode is presented in Fig. 1.

The weaning process in both groups was the same. The ICU in this study was a closed concept ICU without any visitation. Usually, fast tract extubation is planned for all patients and the use of benzodiazepine is limited. After the first respiratory trigger and signs of wakefulness, patients are oriented by expert nurses. They are assured about the completion of their surgery and its desired outcomes. Usually, patients are informed about catheters and some symptoms such as pain and thirst. After being informed, the weaning process begins and the nurses stand beside the bed for the patients’ assurance. After the endotracheal tube removal, the nurse initiates the oxygen therapy.

### Intervention

For procedure development in the first step, a comprehensive literature review was done. Two expert panels were then held with six experienced specialists (including two critical care specialists, a cardiac surgeon, and three nurses), each lasting for 2 h. Consequently, the following protocol was suggested.

In the experimental group, 1 day before surgery, the family member who was preferred by the patient was taught about the conditions of the ICU ward and the patient (surgery, endotracheal tube, mechanical ventilation, drains, catheters, etc.). Also, they were oriented again just before entering the ICU. The rules regarding the presence of a family member in the ward were as follows: (1) a comfortable place near the patient’s bed was provided for the family member which interfered with neither the patient nor the treatment team. (2) The family member could only care about her/his patient’s condition and was not allowed to interfere in the care and treatment of other patients. (3) The family member must have had full coordination with the treatment team and had to leave the ward on request.

The researcher stood near the patient and checked the whole process for any possible protocol deviation.

After the first respiratory attempt, the family member was requested to talk to his/her patient and explained how successful the surgery was. The family member was encouraged to touch the patient and give him/her assurance about the recovery process. Both the family member and the patient were supported by the nurse. After this step, the family member was a bedside patient until the end of the weaning process. When the patient was fully prepared for extubation, the family member was asked to leave the ward.

In the control group, only routine measures were performed. Based on the routine policy of the hospital, family visitation was not allowed.

### Data collection

The primary outcome of this study was agitation which was measured by RASS. Also, anxiety was assessed using the Faces Anxiety Scale (FAS). Both RASS and FAS were assessed in seven consecutive steps, including the time of entering the ICU, the first respiratory drive, the family entrance, 20 min and an hour after the family presence, the time of extubation, and 1 h after extubation. Different time points were suggested, but finally, the expert panel agreed on these time intervals.

The FAS, as a valid and reliable tool, has been developed by McKinley et al. (Gustad et al. 2005). It has a Likert-based scoring ranging from 1 to 5. Number 1 (no anxiety), number 2 (mild anxiety), number 3 (moderate anxiety), number 4 (severe anxiety), and number 5 (very severe anxiety). There is no need for the patient to speak or participate or respond to the questions in this tool; therefore, it is mostly used in the ICU and in patients under mechanical ventilation.

The RASS was used to measure sedation and agitation. It can be assessed in 30 to 60 s using three consecutive steps: observation, response to auditory stimuli, and response to painful stimuli. This scale is scored from −5 to +4. The zero point is for alert and calm. Positive scores are for agitation ranging from +1 (restless) to +4 (combative). The negative scores are for sedation, ranging from −1 (drowsy) to −5 (unarousable). The Persian version of this scale is valid and reliable (Tadrisi et al. 2009).

By using a simple rating scale, families were requested to describe their feeling about being present in the ICU. The score zero was for the worst and 10 was for the best feelings. After the completion of sampling, nurses were requested to give their scores for the feasibility of interventions and their feelings about family presence. Totally, 25 nurses completed these questions. All measurements were done by the main researcher of the study who was present bedside patients during the sampling process.

### Statistical analysis

Continuous variables are described using mean ± standard deviation (SD) values, and categorical variables are presented as frequency rates and percentages. For comparing the demographic characteristics and baseline measures between the two groups of this study, the independent *t* test for continuous variables and chi-square test or Fisher’s exact test (in case of the low sample) for categorical variables were used. The normality of the numeric variables was tested by Kolmogorov-Smirnov test. The repeated measures analysis of variance (RMANOVA) was used to compare the FAS and RASS measurements within the groups. Also, pairwise comparisons were done by Sidak post hoc test. The assumption of sphericity was addressed by Mauchly’s test of sphericity, and when the assumption was not satisfied (<0.05), the Greenhouse-Geiser correction of *P* value was utilized. Data was analyzed using the Statistical Package for the Social Sciences (SPSS) 21.0 (Chicago, IL, USA) and two-side *P*<0.05 indicated a statistically significant difference.

## Results

**Fig. 2:**
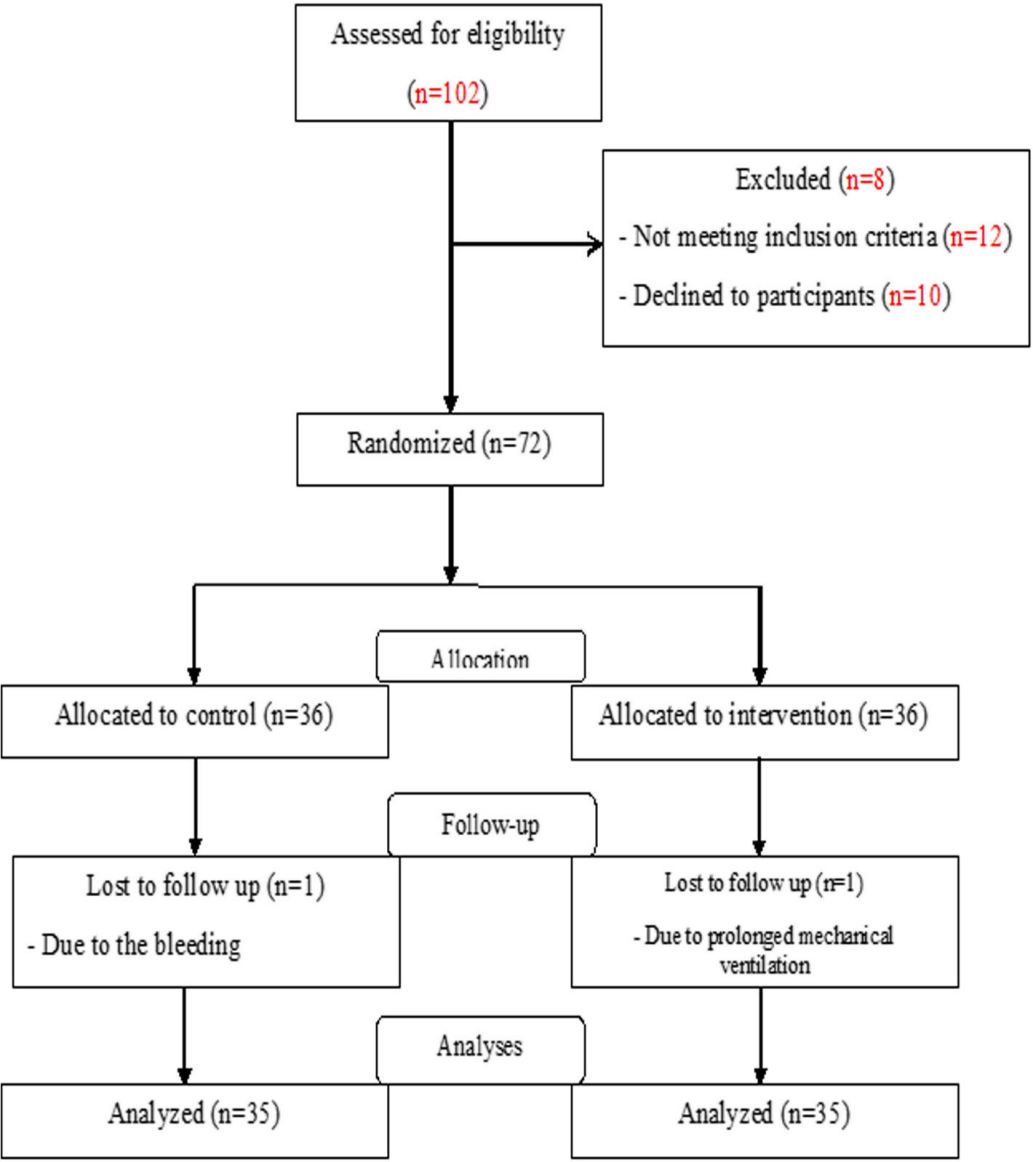
The study flow chart

Totally, 72 patients were enrolled in this study according to the inclusion and exclusion criteria. Among these, two patients were excluded due to excessive bleeding and prolonged mechanical ventilation. Finally, 70 patients (35 in each of the intervention and control groups) completed the study, and analysis was done for these patients. Figure 2 shows the patients’ enrolment flow diagram.

The mean ± SD age of intervention and control groups’ participants was 62.17±9.72 and 62.00±9.17, respectively. The difference between groups was not statistically significant (*P*=0.94). In terms of gender, 10 (28.6%) participants from the intervention group and 8 (25%) from the control group were female. The difference was not statistically significant (*P*=0.73). Other basic demographic data, disease history, and intraoperative data are presented in Table 1. There were no significant differences between groups (*P*>0.05).

**Table 1:** Demographic, pre- and postoperative data

Variable	Intervention group	Control group	P value
Age (mean±SD) (years)	**62.17±9.72**	**62.00±9.17**	**0.94**
BMI (mean±SD) (kg/m^2^)	28.16±4.57	27.88±3.75	0.78
Male/female	25/10	27/8	0.73
Cardiopulmonary bypass time (mean±SD) (min)	65.42±26.50	61.00±24.92	0.49
Intra operative time (mean±SD) (min)	243.28±41.11	237.78±37.00	0.60
Number of smokers	8	7	0.81
Number of opium addicts	8	3	0.18
Ejection fraction (mean±SD) (%)	49.00±7.84	46.53±7.82	0.19
Diabetic/no diabetic	13/22	12/23	0.92
HTN/no HTN	13/22	16/19	0.39

*BMI* body mass index, *HTN* hypertension

Table 2 shows the frequency and percentage of FAS status, in the groups of experimental and control in measurement stages. Man-Whitney *U* test showed a difference between the groups in the mean scores of the anxiety scale in the first (*P* =0.008), second (*P*=0.002), and third stages (*P* =0.005). This difference was not significant in other measurement stages (*P*>0.05). As the baseline anxiety levels were different, the covariate adjustment was used for comparisons between treatments, adjusting the main analyses for baseline anxiety levels. Analysis showed that the different groups were not different. Repeated measurements ANOVA showed that the trend of changes during the seven stages of measurement was significant both within and between groups (*P*<0.001). The comparison of RASS during the seven stages did not show any significant difference between the two groups of experimental and control (Table 3).

**Table 2:** Comparison of anxiety status at different measuring times

	Group	Description	Frequency (%)						
			First step	Second step	Third step	Fourth step	Fifth step	Sixth step	Seventh step
**Faces anxiety scale**	**Experimental**	No anxiety	**(77.1) 27**	**(62.9) 22**	**(62.9) 22**	**(62.9) 22**	**(77.1) 27**	**(34.3) 12**	**(94.3) 33**
		Mild anxiety	**(17.1) 6**	**(33.4) 12**	**(24.3) 12**	**(37.1) 13**	**(20.00) 7**	**(48.6) 17**	**(5.7) 2**
		Moderate anxiety	**(5/7) 2**	**(2.9) 1**	**(2.9) 1**	**0**	**(2.9) 1**	**(11.4) 4**	**0**
		Severe anxiety	**0**	**0**	**0**	**0**	**0**	**(5/7) 2**	**0**
		Very severe anxiety	**0**	**0**	**0**	**0**	**0**	**0**	**0**
	**Control**	No anxiety	**(44.4) 16**	**(25.00) 9**	**(30.6) 11**	**(44.4) 16**	**(61.1) 22**	**(19.4) 7**	**(83.3) 30**
		Mild anxiety	**(47.2) 17**	**(69.4) 25**	**(58.3) 21**	**(44.4) 16**	**(36.1) 13**	**(47.2) 17**	**(16.7) 6**
		Moderate anxiety	**(8.3) 3**	**(5.6) 2**	**(11.1) 4**	**8.3)) 3**	**(2.8) 1**	**(27.8) 10**	**0**
		Severe anxiety	**0**	**0**	**0**	**(2.8) 1**	**0**	**(2.8) 1**	**0**
		Very severe anxiety	**0**	**0**	**0**	**0**	**0**	**((2.8) 1**	**0**
**Significant level of steps**			**P=0.008**	**0.002** = P	**0.005** = P	**0.064** = P	**0.164** = P	**0.084** = P	**0.147** = P
**Significant level within the intervention group**			0.001>*P*						
**Significant level within the control group**			0.001>*P*						
**Significant level between groups**			0.001>*P*						

The measured steps are as follows: the time of entry into the ICU, the first respiratory drive, the family entrance, 20 min after the family presence, and 1 h after the presence of the family member, the time of extubation, and 1 h after extubation

**Table 3:** Comparison of RASS at different measuring

	Group	Description	Frequency (%)						
			First step	Second step	Third step	Forth step	Fifth step	Sixth step	Seventh step
**Richmond Agitation Sedation Scale**	**Experimental**	**Combative**	**0**	**0**	**0**	**0**	**0**	**0**	**0**
		**Very agitated**	**0**	**1 (2.9)**	**1 (2.9)**	**0**	**0**	**0**	**0**
		**Agitated**	**0**	**7 (2 0. 0 0)**	**6 (17.1 0)**	**3 (8.6)**	**5 (14.3)**	**0**	**0**
		**Restless**	**0**	**3 (8.6)**	**1 (2.9)**	**5 (14.3)**	**3 (8.6)**	**2 0 (57.1)**	**0**
		**Alert and calm**	**0**	**2 (5.7)**	**9 (25.7 0)**	**1 0 (28.6)**	**21 (6 0. 0 0)**	**14 (4 0. 0 0)**	**34 (97.1)**
		**Drowsy**	**0**	**0**	**4 (11.4)**	**6 (17.1 0)**	**3 (8.6)**	**1 (2.9)**	**1 (2.9)**
		**Light sedation**	**1 (2.9)**	**4 (11.4)**	**4 (11.4)**	**4 (11.4)**	**2 (5.7)**	**0**	**0**
		**Moderate sedation**	**0**	**3 (8.6)**	**4 (11.4)**	**7 (2 0. 0 0)**	**1 (2.9)**	**0**	**0**
		**Deep sedation**	**0**	**12 (34.3)**	**3 (8.6)**	**0**	**0**	**0**	**0**
		**Unarousable**	**34 (97.1)**	**3 (8.6)**	**3 (8.6)**	**0**	**0**	**0**	**0**
	**control**	**Combative**	**0**	**0**	**0**	**0**	**0**	**0**	**0**
		**Very agitated**	**1 (2.8)**	**0**	**1 (2.8)**	**0**	**0**	**0**	**0**
		**Agitated**	**0**	**8 (22.2)**	**7 (19.4)**	**8 (22.2)**	**4 (11.1)**	**0**	**0**
		**Restless**	**0**	**1 (2.8)**	**2 (5.6)**	**4 (11.1)**	**4 (11.1)**	**25 (69.4)**	**1 (2.8)**
		**Alert and calm**	**0**	**2 (5.6)**	**4 (11.1)**	**7 (19.4)**	**18 (5 0. 0 0)**	**9 (25. 0 0)**	**32 (88.9)**
		**Drowsy**	**0**	**0**	**4 (11.1)**	**1 0 (27.8)**	**1 0 (27.8)**	**2 (5.6)**	**1 (2.8)**
		**Light sedation**	**0**	**3 (8.3)**	**1 (2.8)**	**2 (5.6)**	**0**	**0**	**0**
		**Moderate sedation**	**0**	**5 (13.9)**	**12 (33.3)**	**3 (8.3)**	**0**	**0**	**0**
		**Deep sedation**	**1 (2.8)**	**13 (36.1)**	**5 (13.9)**	**2 (5.6)**	**0**	**0**	**0**
		**Unarousable**	**33 (94.4)**	**4 (11.1)**	**0**	**0**	**0**	**0**	**0**
**Significant level of steps**			***P*= 0.57**	***P*= 0.56**	***P*= 0.65**	***P*= 0.36**	***P*= 0.58**	***P*= 0.35**	***P*= 0.18**
**Significant level within the intervention group**			*P*<0.001						
**Significant level within the control group**			*P*<0.001						
**Significant level between groups**			*P*= 0.94						

The measured steps are as follows: the time of entry into the ICU, the first respiratory drive, the family entrance, 20 min after the family presence, and 1 h after the presence of the family member, the time of extubation, and 1 h after extubation

The mean score of families’ feelings about their presence was 7.05±1.71. Nurses scored the feasibility of the family presence and their feeling about the family presence 7.32±1.95 and 7.36±1.42, respectively.

## Discussion

This study was conducted in order to determine the effect of the family presence on anxiety and agitation during the acute phase after cardiac surgery. Results revealed that anxiety levels did not decrease after the family presence in the intervention group.

Reduction of anxiety after the family presence has been reported in different contexts including burn, myocardial infarction, and ICU (Black et al. 2011; Fumagalli et al. 2006; Lolaty et al. 2014; Bishop et al. 2013). Other studies have not reported any benefits from the family presence (İşlekdemir and Kaya 2016; Sağlık and Çağlar 2019).

The emotional connection of family members and their patients can lead to a better acceptance of the disease and proper transmission of information. We had this hypothesis that providing the postoperative explanation by family members (in addition to medical staff) could help in anxiety reduction and better control of physiological responses. In other words, could help the patient to cope with the stress, before entering the stress phase.

The family presence is considered as part of the treatment process in the neonatal and pediatric wards (Khajeh et al. 2017). In other sections, however, especially in ICUs, visitation is a controversial concept. In recent years, positive effects of the visits are documented, and the family presence is considered as a caring philosophy. The spectrum of family roles ranges from simple presence to participation in therapeutic processes and, at a higher level, decision making. In Iran, the concept of family participation is generally described as delegating some of the patient’s basic caring processes to the family, and families are not well involved in decision-making. Participation has two important prerequisites: education and supervision. Families should be educated and their function and participation must be monitored (Moradian et al. 2017).

We found that family presence was not effective in reducing agitation. The trend of RASS changes revealed that with the passage of time and the recovery from anesthesia, patients in both groups moved from drowsiness to consciousness. The comparison between groups did not show a significant difference in agitation trends. The results also showed that only a small number of patients developed severe agitation, and none of the patients were combative. It can be concluded that in the initial phase after surgery, the prevalence of anxiety is higher than agitation. Therefore, this condition may require further interventions. Clinical variables have been reported as the main predictors of agitation in adult critically ill patients (Burk et al. 2014).

There are different styles of family visits, ranging from limited presence to liberal visitation at all hours (Liu et al. 2013). Also, the presence of family members in the acute phase of the disease is often challenged, and most studies consider the presence of family members only in the inactivity of therapeutic interventions (Cappellini et al. 2014; Colbert and Adler 2013). In this study, family visits were planned during the time that most interventions including mechanical ventilation, weaning, hemodynamic monitoring, recovery from anesthesia, control of bleeding, and patient orientation were performed. The presence of families in the acute phase of the disease was described as feasible by nurses. In the study of Jabre et al. (2013), the presence of a family member during cardiopulmonary resuscitation was associated with positive psychological effects (Jabre et al. 2013). However, some studies have found that open visits increase workload and delays care (Biancofiore et al. 2010).

### Limitation

As visits were prohibited by the hospital’s policies, visitations were allowed for limited hours. The positive effects of the family presence could be more evident in the prolonged presence of family members.

The baseline anxiety levels were different between groups in steps 1–3. Data shows that most of the cases either had no anxiety or mild to moderate anxiety. During these steps, none of the patients had severe or very severe anxiety. We tried to control this effect using covariate adjustment for comparisons between treatments, adjusting the main analyses for baseline anxiety levels. Also, there is a possibility to have a chance of finding related to the relatively small sample size and the limitations of the accuracy of the measurements used.

## Conclusion

According to findings, it seems that the family presence does not reduce the level of anxiety and agitation of patients undergoing cardiac surgery. Nurses described this intervention as feasible. Thus, the decision regarding the family presence for reducing anxiety and agitation should be based on the preference of both the patient and the family member.

## Data Availability

All published data is available and could be presented if requested.

## Abbreviations

CABG: Coronary artery bypass graft surgery
ICU: Intensive care unit
FiO_2_: Friction of inspired oxygen
ABG: Arterial blood gas
ASV: Adaptive support ventilation
FAS: Faces Anxiety Scale
RASS: Richmond’s Anxiety Sedation Scale
SD: Standard deviation
RMANOVA: Repeated Measures Analysis of Variance
